# Treating Proximal Tibial Growth Plate Injuries Using Poly(Lactic-co-Glycolic Acid) Scaffolds

**DOI:** 10.1089/biores.2014.0034

**Published:** 2015-01-01

**Authors:** Amanda Clark, J. Zach Hilt, Todd A. Milbrandt, David A. Puleo

**Affiliations:** ^1^ Department of Biomedical Engineering, University of Kentucky, Lexington, Kentucky.; ^2^ Department of Chemical and Materials Engineering, University of Kentucky, Lexington, Kentucky.; ^3^ Department of Orthopedic Surgery, Mayo Clinic, Rochester, Minnesota.

**Keywords:** growth plate, insulin-like growth factor I, physeal injury, poly(lactic-co-glycolic acid), scaffold

## Abstract

Growth plate fractures account for nearly 18.5% of fractures in children. Depending on the type and severity of the injury, inhibited bone growth or angular deformity caused by bone forming in place of the growth plate can occur. The current treatment involves removal of the bony bar and replacing it with a filler substance, such as a free fat graft. Unfortunately, reformation of the bony bar frequently occurs, preventing the native growth plate from regenerating. The goal of this pilot study was to determine whether biodegradable scaffolds can enhance native growth plate regeneration following a simulated injury that resulted in bony bar formation in the proximal tibial growth plate of New Zealand white rabbits. After removing the bony bar, animals received one of the following treatments: porous poly(lactic-co-glycolic acid) (PLGA) scaffold; PLGA scaffold loaded with insulin-like growth factor I (IGF-I); PLGA scaffold loaded with IGF-I and seeded with autogenous bone marrow cells (BMCs) harvested at the time of implantation; or fat graft (as used clinically). The PLGA scaffold group showed an increased chondrocyte population and a reduced loss of the remaining native growth plate compared to the fat graft group (the control group). An additional increase in chondrocyte density was seen in scaffolds loaded with IGF-I, and even more so when BMCs were seeded on the scaffold. While there was no significant reduction in the angular deformation of the limbs, the PLGA scaffolds increased the amount of cartilage and reduced the amount of bony bar reformation.

## Introduction

The growth plate is a cartilaginous region of long bones that is responsible for bone growth, which continues until puberty when the influence of hormones causes it to close.^1–3^ About 50% of children 5–18 years old will experience a fracture, usually caused from trauma or playing sports. Of those fractures, an estimated 18% of the them will involve the growth plate, damage to which can result in angular deformation or inhibited growth.^4^ A reported 30–65% of growth plate fractures resulted in some level of growth disturbance, depending on the type and location of the fracture.^5^

Growth plate injuries are so detrimental because the cartilage is unable to regenerate,^6^ which leads to the formation of bone at the place of fracture, referred to as a bony bar (or bridge).^7^ The bony bar acts as a tether that prevents certain areas of the physis from expanding.^8^ To prevent impaired growth, current treatment options involve removal of the bony bar and replacement with fat, muscle, cartilage, polymeric silicone, bone wax, or bone cement as interpositional materials.^9–11^ The Langenskiöld method, which involves removing the bony bridge and replacing it with a free fat transplant, has been shown to be a better alternative to osteotomy and limb lengthening.^12^ However, about 60% of patients who receive this treatment have fair to poor results, and approximately 30% of patients experience a reoccurrence of bony bar formation.^11^

Current research for other treatment options largely surrounds the use of mesenchymal stem cells (MSCs). Li et al. implanted chitin scaffolds cultured with MSCs for 7 days into the medial half of the proximal tibia growth plate of rabbits after excising the bony bar. A significant decrease in angular deformation and longitudinal discrepancy compared to the unoperated tibia was reported.^13^ Similar results were seen in other work involving sodium hyaluronate/collagen or agarose scaffolds where limb length improved.^14–16^ The drawbacks of this approach, however, are that MSCs must first be harvested during an additional surgery as well as the time needed for preculture on the scaffolds.

The objective of the present pilot animal study was to determine whether previously developed poly(lactic-co-glycolic acid) (PLGA) scaffolds loaded with insulin-like growth factor I (IGF-I) enhance growth plate regeneration compared to a currently used clinical treatment, such as a fat graft.

## Materials and Methods

### Scaffold fabrication

Microspheres were fused to form porous PLGA (50:50, molecular weight: 31–58 kDa, acid-terminated; Durect Corporation, Pelham, AL) scaffolds as previously reported.^17^ To fabricate the blank and IGF-I-loaded scaffolds (PeproTech, Rocky Hill, NJ), 120 mg of PLGA microspheres (<250 μm) were mixed with 180 mg of NaCl particles (<150 μm) and compressed in a 6.5-mm-radius die at 7 tons for 2 minutes using a Carver press. The scaffolds were sintered for 48 h at 49°C, leached in deionized water overnight, and dried. The scaffold was cut in half to better fit the implantation site. The final dimensions of all the scaffolds were a 6.5-mm-radius semicircle with 1.2 mm height. The IGF-I-loaded semicircle scaffolds contained 27–35 μg of IGF-I.

For disinfection, each scaffold was placed in a 50 mL centrifuge tube and centrifuged at 200 *g* for 5 min in 5 mL of 70% ethanol followed by two wash steps with sterile PBS. The scaffolds were then dried in a laminar flow hood overnight.

### Animal surgery

**FIG. 1.**
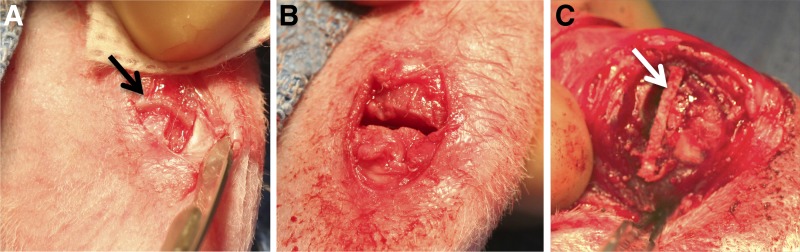
The site of implantation before **(A)** and after **(B)** growth plate removal. The black arrow indicates intact growth plate. **(C)** Trimmed and implanted scaffold (white arrow) following resection of the bony bar.

**FIG. 2.**
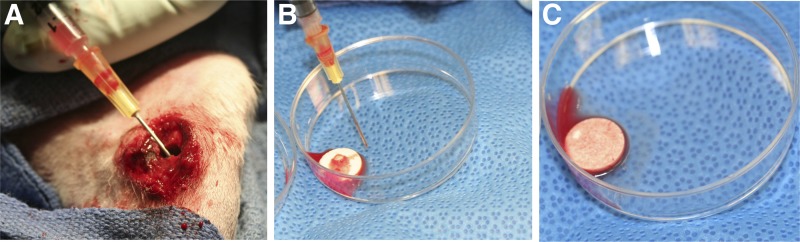
Bone marrow was harvested from the diaphysis **(A)**, seeded on scaffolds **(B)**, and absorbed into the scaffolds for 20 min **(C)**.

All animal studies were conducted at the University of Kentucky in accordance with a protocol approved by the Institutional Animal Care and Use Committee (IACUC). Fifteen New Zealand white female rabbits, 6–8 weeks old, were used. To simulate a growth plate injury,^13^ the medial one-third of the proximal tibial growth plate was removed unilaterally using a 1.0 mm bur (Stryker Medical, Malvern, PA), as seen in Figure 1A and B. The wound was thoroughly irrigated with saline and closed using sutures. Radiographic images were taken to verify location of the growth plate defect. After 3 weeks, radiographs were obtained to confirm formation of a bony bar across the defect. The bony bar was then resected using the same procedure described previously, and an implant trimmed to fit the defect was placed in the site (Fig. 1C). Animals were assigned to one of four treatment groups: (1) fat, removed from the infrapatellar fat pad; (2) blank (without IGF-I) scaffold; (3) IGF-I-loaded scaffold; and (4) IGF-I-loaded scaffold with bone marrow cells (BMCs) harvested at the time of surgery (Fig. 2). An empty defect group was not included in this study because a previous experiment confirmed collapse of the tibial plateau and bridging with bone.^18^ For group 4, after removal of the bony bar, bone marrow was aspirated from the implant site and tibial diaphysis using a syringe, seeded onto the scaffold, and given 20 min for absorption. Upon recovery, the animals were returned to their cages and allowed to move freely with no immobilization. After 8 weeks, the animals were euthanized and another radiograph was obtained.

### Microcomputed tomography

At the end of the study, a 3D reconstruction of the proximal tibia was created using a Scanco μCT40 (Scanco Medical, Zürich, Switzerland). Samples were imaged at 6 μm voxel resolution using scan parameters of 55 kV and 145 mA. The reconstructions were used to qualitatively evaluate the ability of the scaffolds to prevent bone formation in and around the defect area.

### Anatomical measurements

**FIG. 3.**
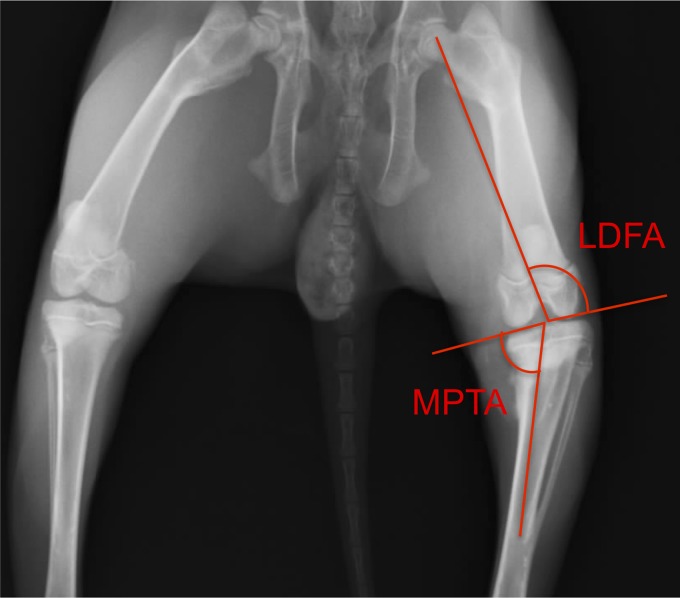
Medial proximal tibial angle (MPTA) and lateral distal femoral angle (LDFA) shown on radiograph.

The medial and lateral lengths of the tibiae and the widths of fibulae were measured for each lower hind limb. Also, using the radiographic images obtained at the time of implantation and euthanasia, the medial proximal tibial angle (MPTA) and lateral distal femoral angle (LDFA) were determined (Fig. 3). All measurements were calculated using ImageJ software.

### Histological analysis

After removal of the fibulae, the proximal tibiae were fixed in 10% buffered formalin for 2 weeks. Flagship Biosciences (Aurora, CO) processed the samples by bisecting the bones using the fibular insertion to the opposite side as reference points and then decalcifying them in 10% formic acid. Coronal sections were cut at 4 μm thickness and stained using hematoxylin and eosin. Stained sections were visualized and photographed using a Nikon Eclipse E600 microscope attached to a Nikon DN 100 digital camera. Observations were based on multiple slides prepared for each specimen.

### Statistical analysis

Analysis was carried out using GraphPad InStat software running an ANOVA followed by a Tukey–Kramer multiple comparisons test. Results were considered significant if *p*<0.05.

## Results

### Postoperative observations

Two of the rabbits became infected after scaffold implantation, both in the IGF-loaded scaffold group. An additional third rabbit in the cell-seeded, IGF-I-loaded scaffold group showed signs of a possible infection but was treated with antibiotics.

### Microcomputed tomography

**FIG. 4.**
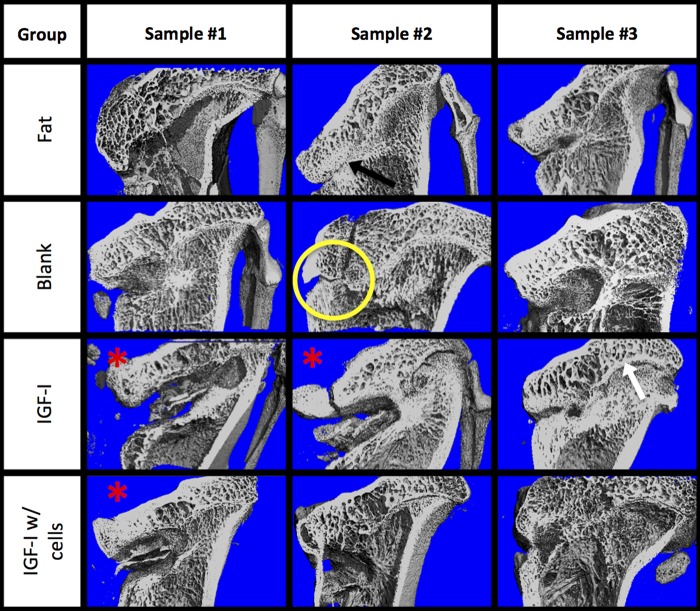
Cut-plane microcomputed tomography images of each tibia for the four treatment groups: (1) fat implant, (2) blank scaffold, (3) IGF-I-loaded scaffold, and (4) cell seeded, IGF-I-loaded scaffold. The defects are on the medial (left) side, and the native growth plate is on the lateral (right) side. The yellow circle indicates the implant site; white arrow indicates the native growth plate; black arrow indicates bony bar formation; red asterisks indicate infected or potentially defects.

A bisected reconstruction of each proximal tibia is shown in Figure 4. Varying amounts of bone growth occurred in all of the noninfected limbs. There appeared to be some bone forming around the implant site in the other animals, especially in the blank and IGF-I-loaded scaffold groups. One example is shown in the yellow circle, indicating the implant site. The black arrow in the figure illustrates a dense bone formation at the implant site, representative of bony bar formation. The small gap on the medial side, where the defect site was created, may indicate a presence of radiolucent cartilaginous tissue.

### Anatomical measurements

**FIG. 5.**
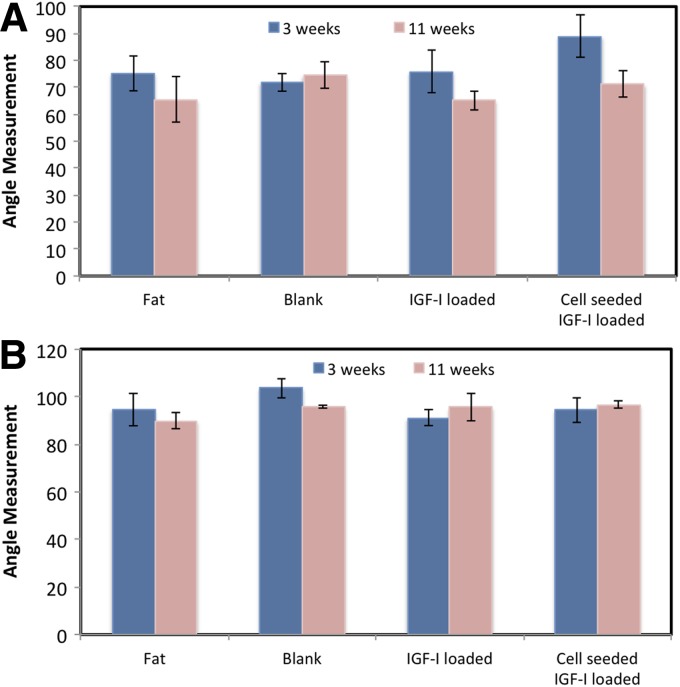
Medial proximal tibial angles **(A)** and lateral distal femoral angles **(B)** at 3 weeks after growth plate injury (before resection of the bony bar) and 8 weeks after implantation of scaffold. Data are shown as means±standard error (*n*≥3).

The MPTA data are shown in Figure 5A. All treatments resulted in an angle decrease 8 weeks after the implantation surgery. The smallest MPTA at 8 weeks was in the IGF-I-loaded scaffold group, which included the two infected animals. The next lowest was the fat implant group, followed by the cell-seeded, IGF-loaded scaffold, and the blank scaffold. No statistical differences were seen between angles at the time of bony bar resection and at 8 weeks following implantation or between scaffold types.

Figure 5B shows the LDFA results, ranging from 90° to 96°, where the fat implant group had the smallest angle, and the cell-seeded, IGF-loaded group had the largest angle at 8 weeks after implantation. The blank scaffold and IGF-I-loaded scaffold groups fell in between, with no statistical difference between any groups.

### Histology

Numerous sections were cut to encompass the full width of the epiphysis at the mid-plane of the tibia. Thus, the region in which scaffolds were implanted was well evaluated and representative results are shown. Neither chronic inflammation nor fibrous tissue was observed in any of the specimens.

**FIG. 6.**
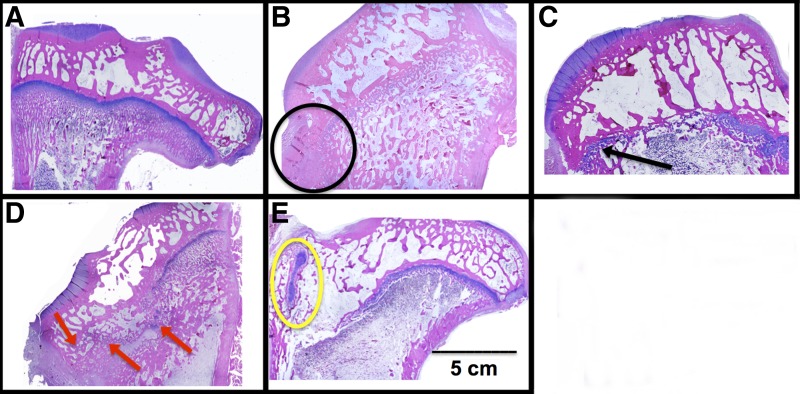
Representative histological images of **(A)** normal proximal tibial growth plate and proximal tibiae following treatment of defects with **(B)** fat implant, **(C)** blank scaffold, **(D)** IGF-loaded scaffold, and **(E)** cell-seeded, IGF-I-loaded scaffold. For images **(B–E)**, the defects were on the medial (left) side, and the native growth plate is on the lateral (right) side. The black circle indicates an area of bone formed within the defect; the black arrow indicates a wide region of newly formed cartilage; red arrows indicate isolated areas of cartilage; and the yellow circle indicates a large, dense area of cartilage.

Figure 6 shows rabbit tibiae sectioned in the coronal plane at 8 weeks after implantation. The remnant of the native growth plate can be seen on the right side of all the images (the lateral side, L), while the implant site can be seen on the left (or medial side, M). The contralateral right legs, which were not injured, served as control (Fig. 6A). A distinct growth plate can be readily seen across the width of the tibia. Defects treated with fat grafts showed a dense region of bone on the medial side where tissue had regrown (Fig. 6B). Compared to the fat graft group, defects treated with a blank scaffold had a wider growth plate across the tibia (Fig. 6C). Treatment with IGF-I-loaded scaffolds, both with and without seeded cells, maintained a structured growth plate on the lateral side and an increased density of chondrocytes on the medial side in areas (Fig. 6D). The addition of BMCs resulted in a large, dense area of cartilage on the upper medial side containing mostly hypertrophic chondrocytes (Fig. 6E).

**FIG. 7.**
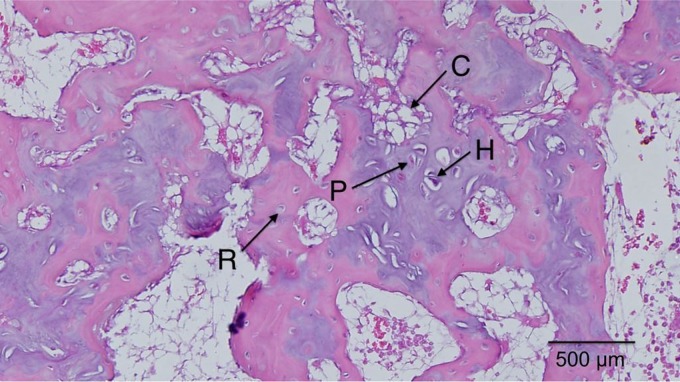
Fat implant showed thin, continual line of cells across medial side that contained reserve (R), proliferative (P), hypertrophic (H) cartilage cells, and calcification zones (C).

**FIG. 8.**
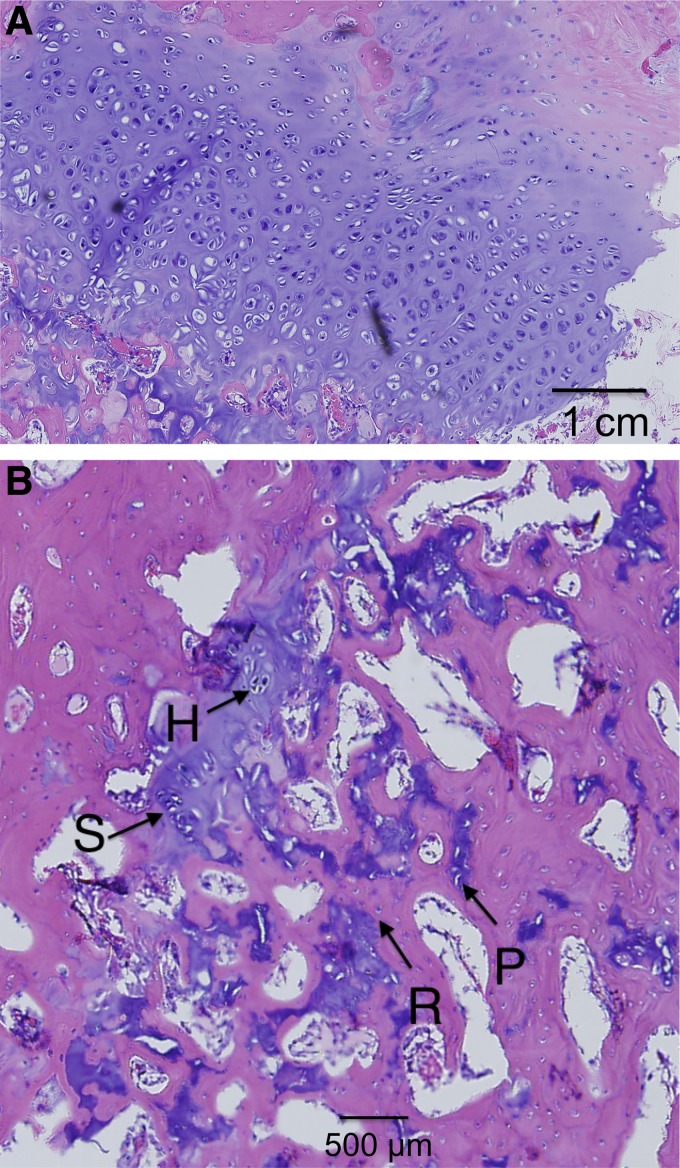
Blank scaffold on **(A)** the lateral side with columnar structure and **(B)** the medial side with the appearance of stacked (S), reserve (R), proliferative (P), and hypertrophic (H) cartilage cells.

**FIG. 9.**
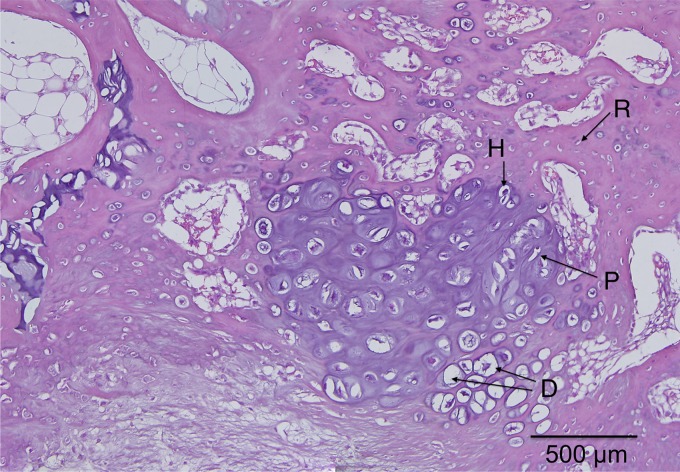
IGF-I-loaded scaffold showed dispersed pockets of cartilage cells throughout the medial side with the appearance of reserve (R), proliferative (P), hypertrophic (H), and degenerative states (D).

**FIG. 10.**
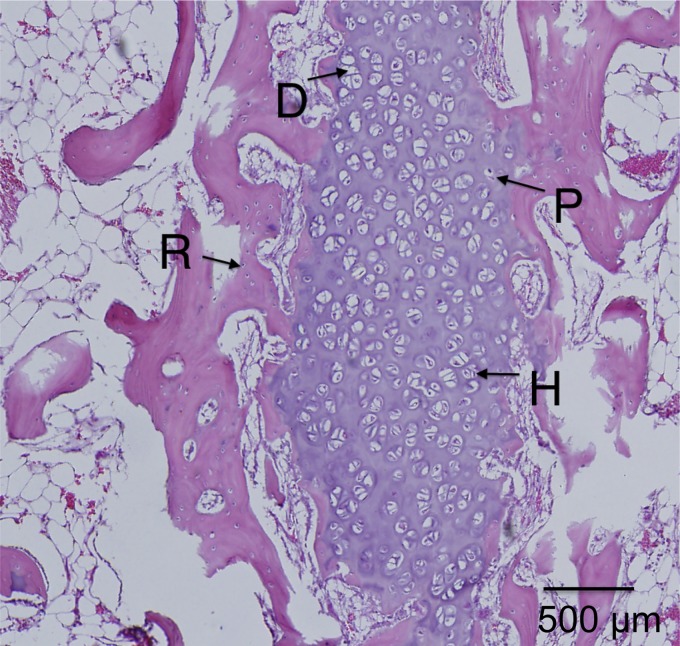
IGF-I-loaded scaffolds with cells showed a large dense population of chondrocytes on the medial side with the appearance of reserve (R), proliferative (P), hypertrophic (H), and degenerative states (D).

Magnified views of selected samples are shown in Figures 7–10, where the specific zones of the chondrocytes (reserve, proliferative, hypertrophic, or calcification zones) could be identified. The reserve (R) chondrocytes were recognized by their smaller size, uniformly spherical appearance. Proliferative (P) chondrocytes were observed more frequently in longitudinal columns with increasing volume of cytoplasm. Hypertrophic (H) chondrocytes zone were identified based on their larger, spherical shape that eventually gave way to nuclear fragmentation as they entered the calcification (C) zone. Figure 7 shows the results of the fat implant with a thin, but continual, connection of cartilage spanning the width of the tibia, where different chondrocytes phenotypes were seen. A defect treated with a blank scaffold had a thicker continual connection of chondrocytes (labeled in Figure 8B) with striations of cartilage below the growth plate area, while the center of the tibia had thin patches of mostly hypertrophic chondrocytes. Treatment with IGF-I-loaded scaffolds resulted in pockets of chondrocytes throughout the medial half of the tibia (Fig. 9). The effect of a cell-seeded, IGF-loaded scaffold is shown in Figure 10, with a large pocket of chondrocytes on the upper medial side containing mostly hypertrophic chondrocytes surrounded by reserve cells (Fig. 10).

## Discussion

This preliminary study was performed to evaluate PLGA scaffolds for regenerating growth plate without using previously harvested MSCs but instead seeding BMCs aspirated from the tibia during the same surgery of implanting the scaffold. The material was selected based on previous research with a lower molecular weight PLGA. Increasing the molecular weight was expected to not only give more structural support to withstand physiologic conditions and thereby allow more time for new growth plate tissue to develop. Additionally, the scaffolds were loaded with IGF-I, which has been shown to increase cell proliferation and increase glycosaminoglycan content, an important cartilage component, production over time.^17^ Compared to the clinically relevant implantation of fat, the use of PLGA scaffolds, with and without IGF-I and BMCs, increased the chondrocytes population and amount of overall cartilage in the epiphyseal plate.

### Polymer and implant selection

In the present study, the molecular weight of the PLGA was ∼30 kDa, which was an increase compared to previous work.^19^ Previously, scaffolds composed of ∼6 kDa PLGA degraded too quickly and allowed collapse of the tibial plateau. Even so, implantation of lower molecular weight PLGA scaffolds resulted in the presence of chondrocytes after 2 weeks with a further increase in rabbits treated with IGF-I-loaded scaffolds. Using a higher molecular weight was postulated to increase structural support to the implant site and allow more tissue regrowth to occur before the scaffold degraded.

PLGA-based scaffolds were chosen because PLGA has been shown to be biocompatible and biodegradable.^20,21^ Drug delivery devices using PLGA microspheres are already FDA approved, such as Lupron Depot, Zoladex, and Atridox, for the treatment of prostate cancer, endometriosis, and fibroids. The mechanical properties of PLGA scaffolds were shown to be suitable for hard and soft tissue implants with an initial compressive modulus of about 100 MPa.^17^

To evaluate the success of a polymeric scaffold for growth plate regeneration, a fat implant was chosen to represent current clinical practice by using the Langenskiöld method in which the bony bar is excised and replaced with transplanted fat.^10,12,22^ Free fat transplants act as a space-filler that slowly undergoes ossification in an attempt to prevent total growth plate closure, but results are fair to poor in ∼60% of patients.^11,23^

Growth factors have been theorized to enhance cartilage regeneration by increasing cell proliferation and decreasing cell apoptosis. Delivery of IGF-I has been shown to enhance bone and cartilage production *in vivo.*^9,24–28^ For this reason, IGF-I was loaded into the PLGA microspheres used to fabricate the scaffolds for this study. Recent investigations demonstrated retention of IGF-I bioactivity during scaffold fabrication, which includes fusion of microspheres at 49°C.^29^ Seeding BMCs onto the scaffold was performed in an effort to add mesenchymal cells to the implant site directly instead of waiting for cell recruitment and proliferation during recovery.

### Anatomical measurements

Growth plate injury causes a decrease in both the MPTA and LDFA; therefore, the larger the angle, the better the recovery. The MPTA and LDFA were both lowest in the fat implant group (regardless of whether the infected animals were included or excluded), and although results were not significant, this may indicate that the fat graft does not correct angular deformation as well as the polymeric scaffolds. Surprisingly, the highest MPTA and second highest LDFA were seen in the blank scaffold group. A similar trend was seen in previous work during an 8-week study, where the blank scaffold group had a higher angle than the other treatment types, although results were not significant.^18^ It should be noted that all measurements were calculated by hand, which contributes to larger errors. Other groups have reported statistical differences, but their studies lasted twice as long as the current study. For example, Planka et al. used MSCs seeded onto sodium hyaluronate/collagen scaffolds in a distal femur model and found a 0.50±0.04 cm increase in bone length and an LDFA lowered by 3.6–4.5° over the control at 4 months following implantation.^16^ Their control was no implant as compared to the clinically relevant fat implant used in the present study. In previous work, an empty control group, which received no implant, resulted in bone formation at the implant site 8 weeks after operation and showed the lowest amount of angular correction.^18^

Eight weeks after implantation, defects treated with BMCs showed a greater presence of chondrocytes, yet no preculture time or additional surgery was required to harvest the cells. Li et al. have shown that chitin-based scaffolds with and without MSCs corrected angular deformation significantly after 16 weeks,^13^ but while Chen et al. reported increased angle recovery and tibial length with MSCs seeded on agarose, agarose alone gave poor results.^14,15^ A major difference between the mentioned scaffolds is their mechanical properties. Agarose, collagen, and hyaluronan are commonly used in tissue engineering but have a low compressive modulus, in the 1–150 kPa range, whereas chitosan and PLGA scaffolds have a compressive modulus of 750 kPa to 100 MPa.^17,30–34^

Within tissue engineering research, PLGA scaffolds with and without growth factors have been shown to create a microenvironment necessary for proliferation and chondrogenic differentiation of MSCs.^11,22,23,35^ Also, the PLGA scaffolds used in this study had a pore size of 50–100 μm, shown to be applicable for cell ingrowth.^36^ Therefore, as long as some of the neighboring native growth plate cells remain viable or MSCs are around the defect site, the scaffold may be adequate to prevent bony bridge formation and allow for growth plate regeneration. Incorporating IGF-I or another type of growth factor into the scaffold may provide the cellular signals necessary to initiate tissue regrowth as discussed later.

Although the angle measurements did not show complete correction of the limb deformation, microcomputed tomography images showed that bone had not completely filled the implant site comparable to the fat implant, the current clinical practice, indicating that bony bar reformation was inhibited. Bony bar formation is one of the major complications associated with growth plate injures.^35,37^ Limb length discrepancy and angular deformation occurred in 58% of distal femur factures involving the growth plate.^5^ Even with the current clinical treatment of fat grafts, bony bridge reformation has been shown to occur nearly 30% of the time.^11^ Johnstone et al. used a type I collagen paste containing osteogenic protein-1 and found promising limb growth until bony bridge formation prevented further growth.^38^

### Histology

The attempt to regenerate the growth plate did not result in layered structure (epiphyseal to metaphyseal zones) to the degree of the native growth plate, regardless of the treatment type. It appeared that the fat implant allowed for some cartilage regeneration, but it was only cell-wide at most points, and most of the chondrocytes had the phenotype of cells in the calcification zone. The tissue surrounding the cartilaginous areas was woven bone, which appears after fractures.^39,40^ The blank scaffold treatment resulted in tissue having a structure similar to that for the fat implant group with a couple exceptions. First, there were areas where the scaffolds were replaced with some cellular stacking, and second, the lateral side retained more structure, resembling that of the native growth plate, compared to defects treated with fat graft. Scaffolds gave the epiphyseal region more structural support, reducing further destruction of the native lateral growth plate.

Defects treated with IGF-I-loaded scaffolds, both with or without seeded cells, showed a similar appearance on the lateral (native group plate) side as that of the blank scaffold group, however, the medial sides were quite different. Without cells, the IGF-I-loaded scaffold resulted in pockets of chondrocytes throughout the medial side along the epiphyseal line that contained cells in all zones of cartilage development. The addition of cells created a large vertical pocket (about 3 mm long) of chondrocytes located in the upper epiphyseal region. Note, however, that interpretation of the IGF-I-loaded samples was limited because only one sample could be used for observation. The cells were mostly in the hypertrophic state and had no columnar organization. Because the immunoinflammatory response to infection will alter wound healing, those specimens were not evaluated histologically. Both types of IGF-I-loaded scaffolds (with and without cells seeding) increased the density of hypertrophic chondrocytes compared to the fat and blank groups. Cells seeded on scaffolds containing IGF-I created the largest population of chondrocytes, which was to be expected based on the literature surrounding IGF-I.^41^ One such example is from Masters et al., who showed that IGF-I mediates chondrogenesis of BMCs through increased cell proliferation and expression of chondrocyte markers.^34^

Certain types of PLA and PLGA implants have been reported to elicit a foreign body response, whether as a result of particulates or acidity generated during degradation.^42,43^ Chronic inflammation and fibrous encapsulation were not observed in the present study, however. The porous nature of the scaffolds allowed exchange to reduce acid buildup, and although an early presence of inflammation cannot be excluded, observation of the implantation sites after complete scaffold degradation showed formation of either cartilage or bone. Additionally, the responses seen in the scaffold groups did not differ from that seen in the autologous fat graft group, which reflects a current clinical treatment.

Compared to the study of Chen et al.,^14^ which lasted for 16 weeks, the histological results for this 8-week study are encouraging, having similarity in the types of cells present, although without columnar structure. Without MSCs, the hyaluronate/collagen, agarose, and chitin scaffolds had either similar hypertrophic pockets of chondrocytes or minimal presence of chondrocytes at all.^13–16^ However, the use of MSCs seeded on the chitin or agarose scaffolds gave more of a columnar structure but had twice as much time to develop compared to samples in the present study.^13^

## Conclusions

The goal of this study was to evaluate the potential of a biodegradable scaffold to enhance regeneration of the growth plate without preculture with MSCs. All types of PLGA scaffolds tested led to better retention of the lateral native growth plate compared to the current clinical treatment of using fat grafts. Incorporating IGF-I into the scaffolds increased the presence of chondrocytes at the defect site. Further enhancement of cartilage formation was observed with the addition of BMCs collected at the time of the surgery to IGF-I-loaded scaffolds. Angular deformation did not recover significantly after 8 weeks regardless of the treatment type, however, the histological results indicated that the necessary cells were present for growth plate regeneration. This study showed that growth factor-loaded PLGA scaffolds were not only as effective as the current fat graft treatment in growth plate regeneration, but they also enhanced the chondrocyte population at the implant site and decreased the destruction of the remaining native growth plate.

## Abbreviations Used

BMCs: bone marrow cells
IGF-I: insulin-like growth factor I
LPTA: lateral proximal tibial angle
MPTA: medial proximal tibial angle
MSCs: mesenchymal stem cells
PLGA: poly(lactic-co-glycolic acid)
